# Estimating the contribution of Helicobacter pylori to gastric cancer

**DOI:** 10.1054/bjoc.2000.1392

**Published:** 2000-09-04

**Authors:** J Danesh

**Affiliations:** Clinical Trial Service Unit and Epidemiological Studies Unit, University of Oxford, Radcliffe Infirmary, Oxford, OX2 6HE, UK


doi: 10.1054/ bjoc.2000.1392, available online at http://www.idealibrary.com on IDEAL®

### Estimating the contribution of *Helicobacter pylori* to gastric cancer

**Sir,**

A synthesis of available prospective epidemiological studies indicates that chronic infection with *Helicobacter pylori* is about two or three times as common in people with gastric cancer than in others (risk ratio 2.5, 95% confidence interval 1.9–3.4) (Danesh, 1999a). As stated previously (Danesh, 1999b), some potential biases in published studies may have exaggerated this estimate (such as the preferential publication of more striking findings and/or inadequate adjustment for possible confounders, e.g., smoking); by contrast, other possible biases may have resulted in some underestimation (such as the use of serological assays in developing countries based on bacterial antigens found in developed countries and/or failure of studies to exclude from analyses the first few years of follow-up to minimize any effects of early gastric cancer on *H. pylori* serostatus) (Danesh, 1999b). Such factors cannot, however, plausibly account for much of the overall association observed between *H. pylori* infection and gastric cancer (although more appropriate allowances for such biases in future studies might somewhat change the present estimate of the magnitude of risk).

Ponce-de-Leon and colleagues discuss another possible bias that might overestimate the association between *H. pylori* infection and gastric cancer. They suggest that, in comparison with non-infected people, *H. pylori* seropositive individuals might receive additional gastroenterological investigation (such as upper gastrointestinal endoscopy) due to increased dyspeptic symptoms, thereby resulting in earlier and more frequent diagnosis of gastric cancer. Any such effect, however, is unlikely to be important, as there is no good evidence of any strong association of *H. pylori* infection with non-ulcer dyspepsia or with other common causes of dyspepsia in the general population, apart from peptic ulceration (Danesh et al, 2000). Moreover, the claim of Ponce-de-Leon and colleagues that there is a higher prevalence of *H. pylori* infection in early gastric cancer than in more advanced disease is of uncertain validity (as their study was based on fewer than 50 non-invasive tumours), and the authors themselves state that there were no significant associations in their study between *H. pylori* status and the interval between symptoms and the diagnosis of cancer.

*J Danesh, Clinical Trial Service Unit and Epidemiological Studies Unit, University of Oxford, Radcliffe Infirmary, Oxford OX2 6HE, UK*

### REFERENCES

Danesh J (1999a) *Helicobacter pylori* infection and gastric cancer: time for mega-trials? *Br J Cancer* **80**: 927–929

Danesh J (1999b) *Helicobacter pylori* and gastric cancer: systematic review of the epidemiological evidence. *Aliment Pharmacol Ther* **13**: 851–856

Danesh J, Lawrence M, Murphy M, Roberts S and Collins R (2000) Systematic review of the evidence on *Helicobacter pylori* and dyspepsia. *Arch Intern Med* **160**: 1192–1198

*© 2000 Cancer Research Campaign*

